# Single-staged in vivo co-transplantation of autologous muscular and urothelial micrografts as a composite tissue tube for urogenital reconstruction

**DOI:** 10.1007/s00383-025-06259-5

**Published:** 2026-01-09

**Authors:** Alexander Guldmann Clausen, Nikolai Juul, Mahboobeh Amoushahi, Oliver Willacy, Magdalena Fossum

**Affiliations:** 1 Laboratory of Tissue Engineering, Faculty of Clinical Medicine, University of Copenhagen, Henrik Harpestrengs Vej 4C, Copenhagen, 2100 Denmark; 2 Division of Pediatric Surgery, Department of Surgery and Transplantation, Rigshospitalet Copenhagen University Hospital, Copenhagen, Denmark; 3 Laboratory of Tissue Engineering, Department of Women’s and Children’s Health, Center of Molecular medicine, Karolinska Institutet, Stockholm, Sweden

**Keywords:** Tissue engineering, Urothelium, Urinary bladder, Muscle, Smooth, Tissue expansion

## Abstract

**Purpose:**

Tissue-engineered grafts conventionally rely on resource-intensive ex vivo cellularization. Perioperative autologous micrografting allows for direct single-staged graft cellularization omitting laboratory-based in vitro propagation. For urogenital reconstruction, where a muscular layer may be desirable, co-transplanting muscular and urothelial micrografts has previously shown to inhibit urothelial micrograft expansion. This study aimed to assess the effects of adding muscular micrografts in a separate compartment in a tubular collagen-based urinary graft.

**Methods:**

Autologous tissue from twelve minipig bladders was used for implanting tubular grafts that were constructed and implanted subcutaneously as a single-staged in vivo procedure. Each animal received four grafts: two containing urothelial micrografts only (urothelial group) and two containing both urothelial and detrusor muscle micrografts (co-transplanted group).

**Results:**

Six weeks post-implantation, 74% of the urothelial group and 64% of the co-transplanted tubular grafts demonstrated luminal pancytokeratin-positive epithelium (*p* = 0.524). Grafts were analyzed histologically and by immunoassays for identification of epithelium (pancytokeratin), differentiated urothelium (uroplakin II), smooth muscle (α-SMA & desmin), inflammation (CD68 & apoptosis assay), and vascularization (CD31^+^ vessels). No significant differences in cellular markers or epithelization were observed between groups.

**Conclusion:**

Our findings indicate that muscular micrografts can be co-transplanted in a separate compartment in a collagen-based tubular graft without inhibiting co-transplanted urothelial micrograft expansion.

**Supplementary Information:**

The online version contains supplementary material available at 10.1007/s00383-025-06259-5.

## Introduction

Reconstructive surgery is sometimes needed when treating congenital or acquired conditions of the urogenital system [1, 2]. This is often achieved by implantation of autologous non-orthotopic gastrointestinal tissue. However, due to the fundamentally different functions of urothelial mucosa and intestinal epithelium, this may lead to various complications, including metabolic abnormalities [3], mucus formation, risk of stone formation [4], and risk of spontaneous bladder perforation when used to reconstruct the bladder [5]. Tissue-engineered grafts compose an appealing alternative, as they may allow for better customization of the tissue to match the transplanted site and potentially decreased risks of donor site morbidities. In particular, the development of urinary tubular grafts may provide specific benefits in the surgical treatment for urinary diversion or hypospadias repair. In the field of tissue engineering, acellular grafts have previously proven inferior to cellular grafts in terms of preventing strictures and scar tissue formation in large-defect reconstructions [6, 7]. Additionally, when used for complex organ reconstructions (e.g. bladder augmentation), different cell types may be needed to properly mimic the physiological function of the native organ tissue. Different approaches for graft cellularization have been studied [8]. However, very few have been clinically implemented, partly owing to high manufacturing costs, safety concerns, and rigorous approval procedures related to ex vivo cell expansion [9, 10].

Single-staged perioperative autologous tissue micrografting bypasses many of the conventional challenges related to ex vivo cell culture and has mainly been evaluated in skin defects’ reconstruction [11]. In a previous study on tissue-engineered urinary tubular grafts, urothelial micrografts from bladder mucosa were successfully expanded subcutaneously in an in vivo porcine model by attaching the micrografts to a silicone catheter with fibrin glue before implantation [12]. However, when co-transplanted with detrusor muscle micrografts in a random mixed manner, the urothelial growth was significantly inhibited. Furthermore, postoperative evaluations indicated a need to improve the scaffold bio-adhesion of the micrografts to support tissue regeneration within a preset 3D tubular construct, as well as a need to increase biomechanical strength to enable successful in vivo implantation and expansion of the micrografts in a future clinical setting.

To overcome these challenges, we have since introduced the Perioperative Layered Autologous Tissue Expansion (PLATE) graft [13–16]. In brief, the PLATE graft is constructed by harvesting autologous micrografts from a donor tissue and embedding them in a mesh-reinforced collagen-based scaffold. The collagen serves both as a bio-adhesive and as a viable substrate for cell-expansion, while the mesh ensures biomechanical strength and proper surgical handling. Importantly, the PLATE graft procedure is technically feasible to perform in a normal operating room as a single-staged intervention by using the patient’s own body as a bioreactor [15]. The PLATE graft methodology has been used to successfully expand urothelial micrografts from bladder mucosa in vivo in a porcine bladder conduit model [15]. In another study, reconstructing the vaginal canal in rabbits, vaginal mucosa micrografts were co-transplanted with smooth muscle micrografts in two separate compartments of the PLATE graft [17]. Although there were advantageous effects on extracellular matrix formation both macro- and microscopically when including micrografts compared to acellular grafts, the study was not designed to distinguish specific effects on the mucosa when adding muscular micrografts. We perceive the muscular transplants as histological entities containing smooth muscle cells, mesenchymal stem cells, capillary endothelial cells and fibroblasts. As each cell type could be beneficial for extracellular matrix regeneration, we hypothesized that if we could add muscular micrografts to the composite scaffold without inhibiting the expansion of urothelial cells, an increased presence of smooth muscle could have a positive impact on tissue regeneration, neo-vascularization, and result in less inflammation after implantation and take of transplant.

To further explore and optimize the composition of the PLATE graft, before translation in a clinical setting, the present study aimed to examine the impacts of co-transplanting bladder detrusor muscle and urothelial micrografts and to compare these with urothelial micrografting alone. A standardized in vivo model was used by placing the autologous tubularized PLATE grafts in the abdominal subcutaneous space in a porcine animal model.

## Methods

### Compliance with ethical standards

The animal study was performed after ethical permission from the Danish Ministry of Food and Agriculture (ref. no. 2022-15-0201-01206). The experiments were performed at an accredited experimental facility in accordance with the European legislation on laboratory use of animal subjects (AAALAC International). The study was reported in adherence with the ARRIVE guidelines [18].

### Experimental design

**Fig. 1:**
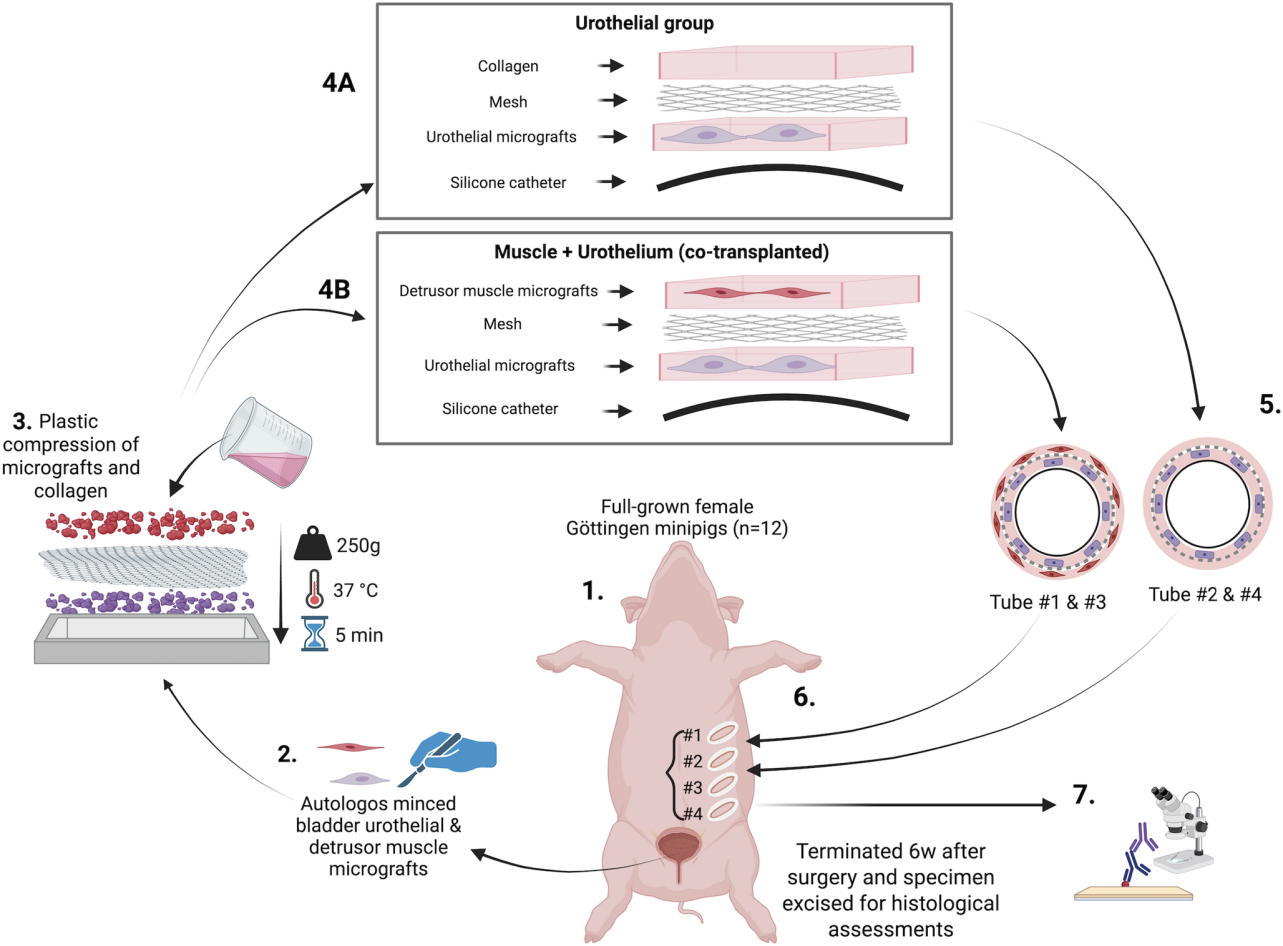
Schematic illustration of the experimental setup. Bladder tissue was harvested from 12 full-grown female minipigs (1) and minced into mucosal and detrusor muscle micrografts, respectively (2). After seeding of the micrografts, the mesh was embedded in liquid collagen 1, followed by consolidation and plastic compression (3) For tube #1 & #3 (co-transplanted), the mucosal and muscle micrografts were seeded on each side of the surgical mesh (4B), and for tube #2 and #4 (urothelial group) mucosal micrografts were placed on one side of the surgical mesh (4 A). The grafts were then sutured around a silicone catheter (5), with urothelial micrografts facing the lumen, before being implanted back subcutaneously into the pig in an alternating order (6). The pigs were terminated after 6 weeks, and the excised graft specimens were fixed for histological assessments (7))

The experimental setup is presented in Fig. 1. Each animal was used as its own control, comparing either implanted urothelial micrografts only (urothelial group) or co-transplanted urothelial micrografts and detrusor muscle micrografts (co-transplanted group). Since the potential future applications of the PLATE graft include reconstruction of tubular organs, such as the urethra and ureters, the PLATE graft was tubularized around a silicone catheter.

**Fig. 2:**
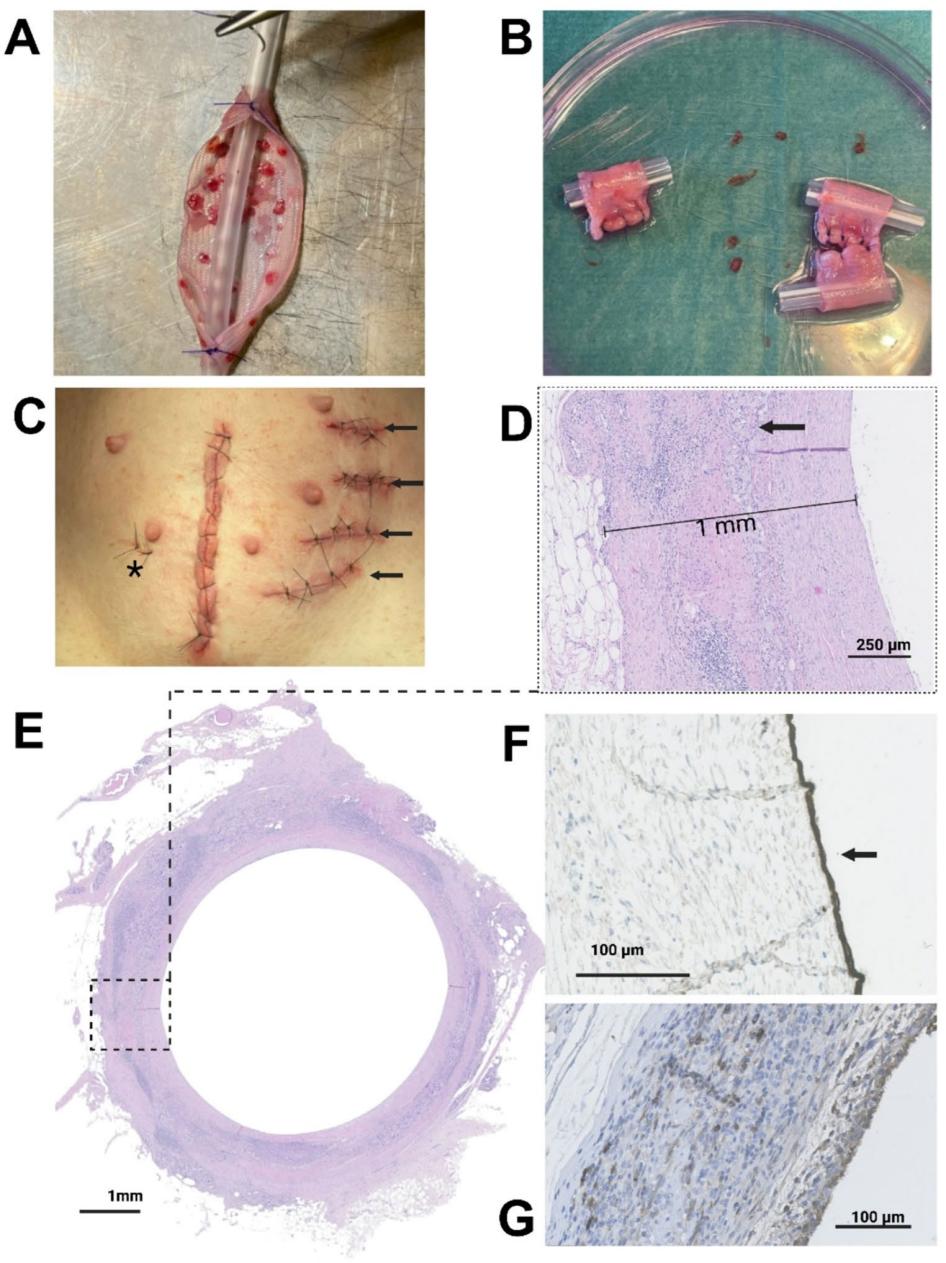
Graft implantation and histological evaluation. **A** The perioperatively constructed graft is sutured around a silicone stent. Tissue micrografts can be seen embedded in collagen on top of a surgical mesh. **B** The grafts are cut into smaller pieces and **C** implanted laterally to the mamillary papillae. Arrows indicate placement of the grafts. Asterix indicate sutures related to a different study. The incision from the midline laparotomy used to harvest bladder and muscle micrografts can also be seen. **D** The thickness of the graft, from the lumen to the subcutaneous adipose tissue, is approximately 1 mm. The arrow indicates visible remnants of the mesh near the center of the graft wall. Numerous cell nuclei are visible around the mesh. **E** Cross-sectional H&E staining of a co-transplanted graft 6 weeks post implantation. **F** Pancytokeratin immunohistochemical staining of a cross-section of a graft containing mucosal micrografts only (urothelial group). Arrow indicates a layer of 1–2 positive cells luminally. **G** Uroplakin II staining of a graft containing mucosal micrografts (urothelial group) to evaluate the presence of differentiated urothelial cells

**Fig. 3:**
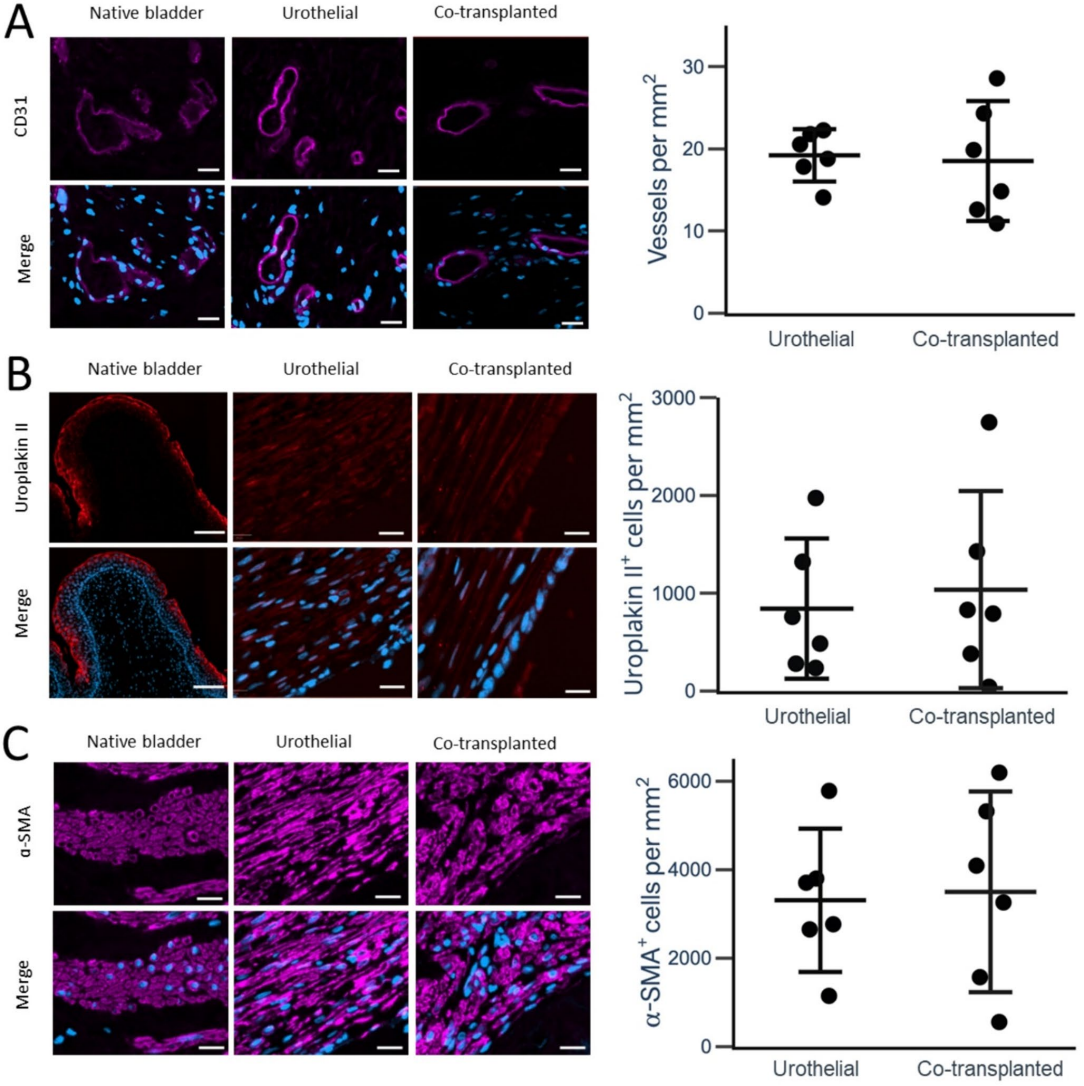
Immunofluorescence microscopy images and quantification of CD31-positive vessels, uroplakin II-positive cells, and α-SMA-positive cells. For all graphs, the horizontal bars indicate means with whiskers indicating 95% confidence interval of the means. **A** CD31 staining (pink) with and without DAPI nuclear staining to show vessels in native bladder, urothelial group, and co-transplanted group. No significant difference between the two groups was observed. Scale bars indicate 20 μm. **B** Uroplakin II stains (red) with and without DAPI nuclear staining in native bladder, urothelial group, and co-transplanted groups. No significant difference between the two groups was observed. Scale bars indicate 100 μm in the native bladder image and 20 μm in the urothelial and co-transplanted group. **C** α-SMA (pink) stains with and without DAPI nuclear staining (blue) in tissue sections of native bladder, urothelial group, and co-transplanted group. No significant difference between the two experimental groups was observed. Scale bars indicate 20 μm

**Fig. 4:**
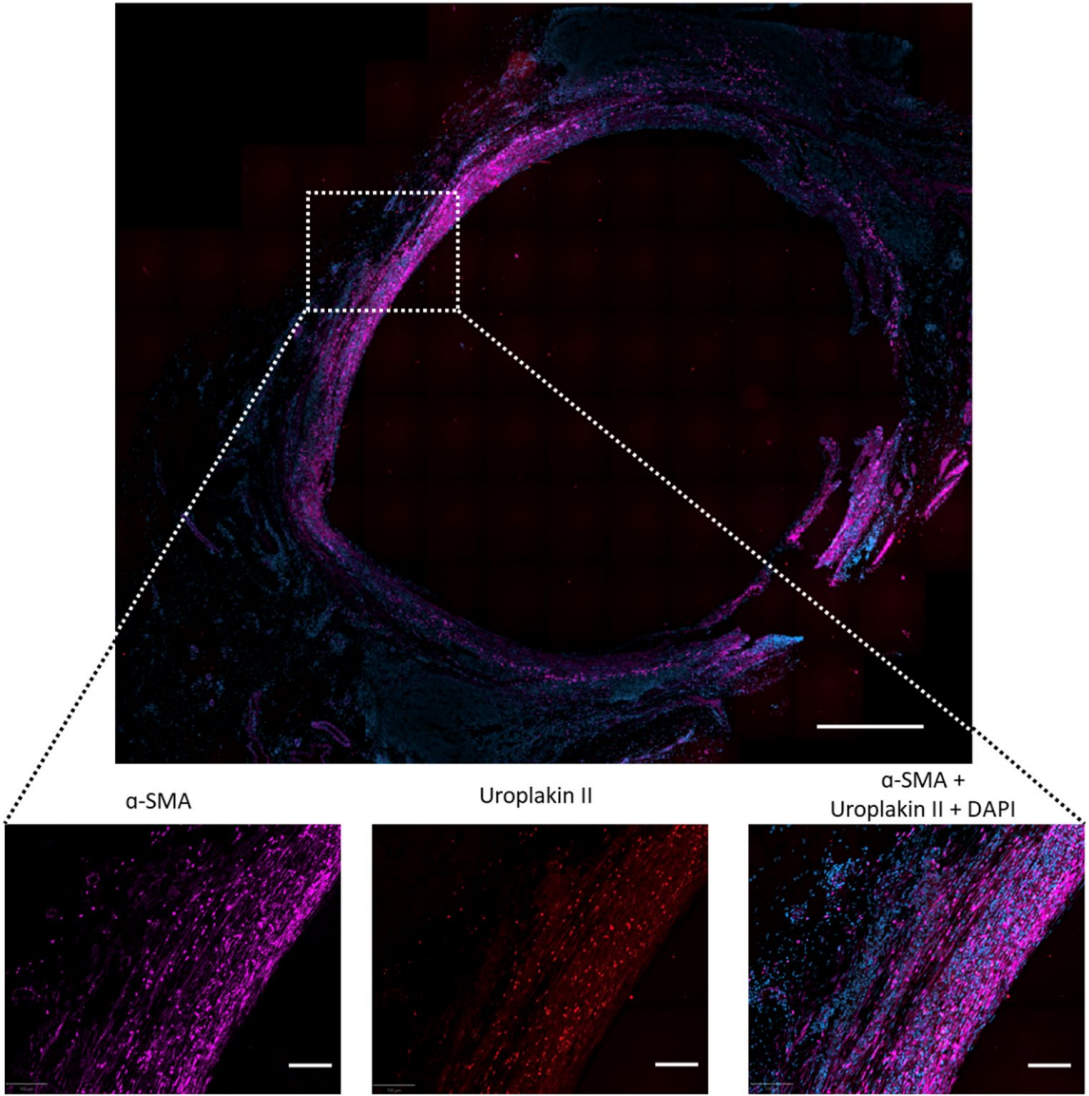
Distribution of uroplakin II & α-SMA in a urothelial micrografted scaffold (urothelial group). Top picture shows immunofluorescence microscopy images of α-SMA (pink), uroplakin-II (red), and DAPI nuclear staining (blue). In the bottom three pictures, possible co-localization of α-SMA & Uroplakin-II is shown. In the top picture scale bar indicates 500 μm and in the bottom pictures scale bars indicate 40 μm

**Fig. 5:**
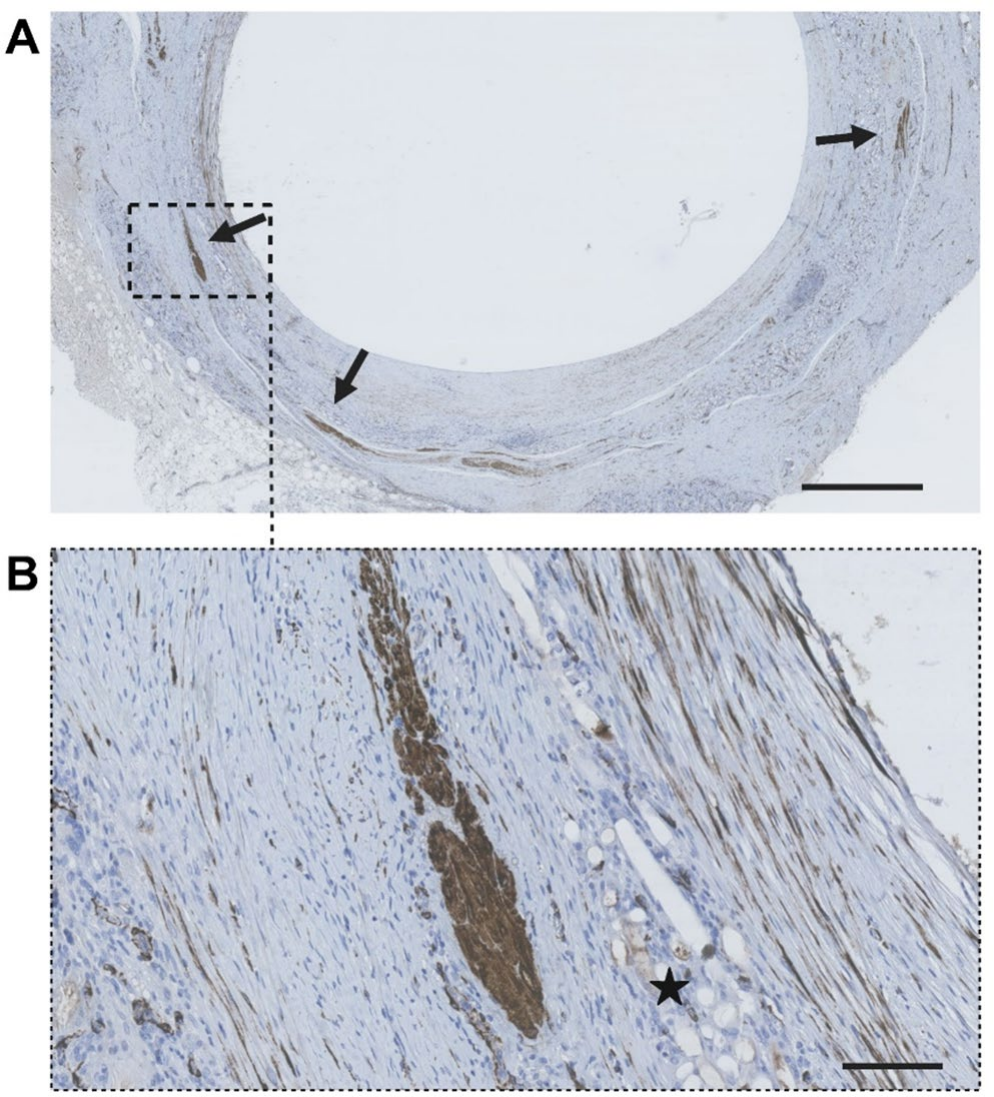
Immunohistochemical staining of desmin (brown) in a co-transplanted graft with hematoxylin counterstain. **A** Desmin-positive areas in the graft wall are indicated with arrows. Scale bar indicates 1 mm. **B** A zoomed in image of a desmin positive area. Star indicates mesh remnants. The desmin positive area is seen to be located on the non-luminal side of the mesh, similarly to where the muscular micrografts were placed at graft construction. Scale bar indicates 100 μm

**Fig. 6:**
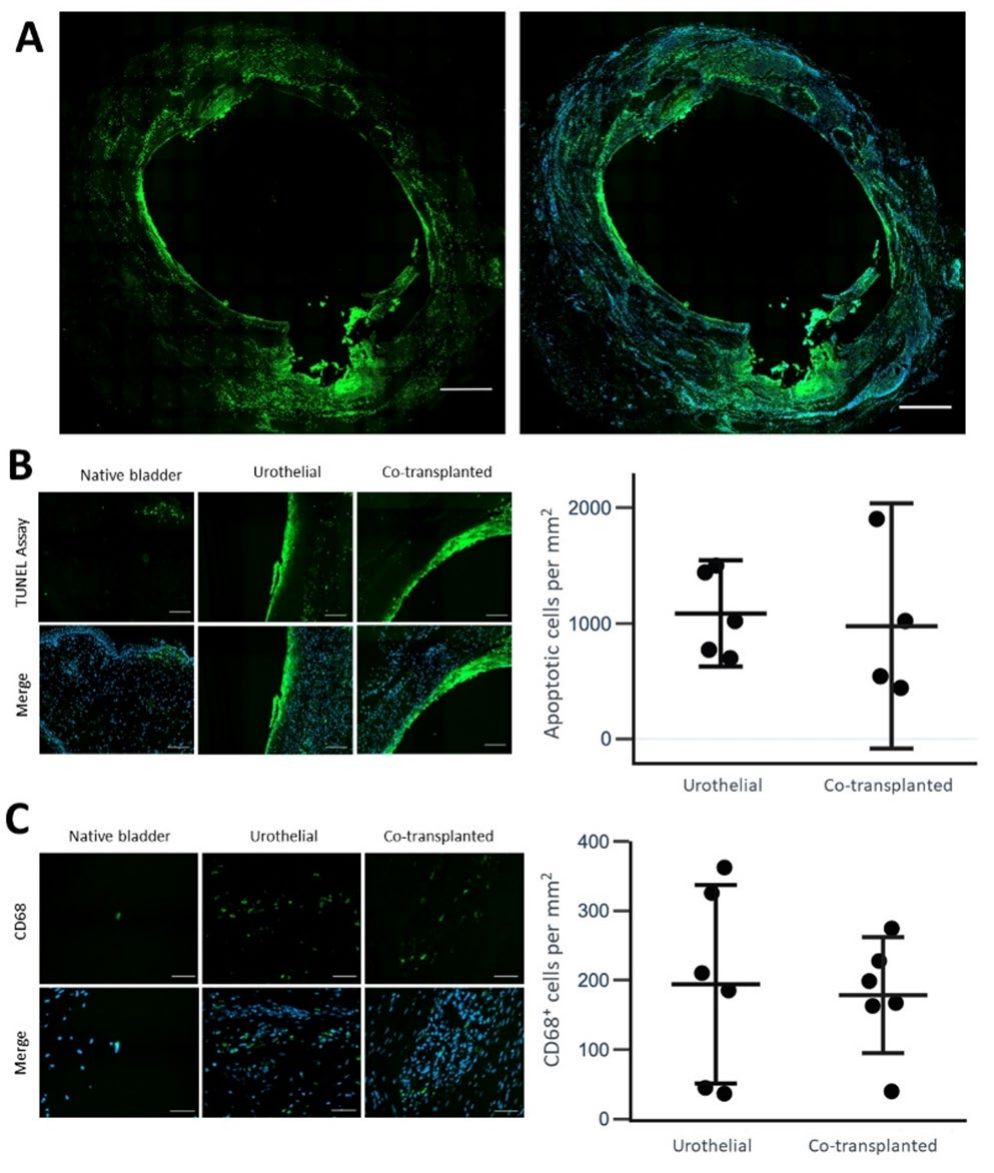
Apoptotic activity and distribution of CD68^+^ cells. For all graphs, the horizontal bars indicate means with whiskers indicating 95% confidence interval of the means. **A** Fluorescence microscopy images of TUNEL assay (green), indicating apoptosis, with DAPI nuclear staining (blue) in the right image. The imaged graft is a co-transplanted graft. Apoptotic activity is primarily observed close to the mesh remnants and towards the lumen. Scale bars indicate 800 μm. **B** Fluorescence microscopy images of TUNEL assay of native bladder, urothelial group, and co-transplanted group. Scale bars indicate 100 μm. No significant difference of TUNEL activity is seen between the two groups. **C** Immunofluorescence microscopy images of CD68^+^ cells in native bladder, urothelial group, and co-transplanted group. Scale bars indicate 50 μm. No significant difference of the number of CD68^+^ cells is seen between the two

### Surgery

Twelve full-grown female Göttingen minipigs, averaging one year of age and weighing 35–45 kg, were included. In accordance with the 3R principles of animal research [19], the pigs were also used for a different non-related experiment that also required bladder tissue, which was performed during the same intervention to reduce the number of research animals [15]. For the current experiment, the methods of sedation, intubation, and surgical preparation were performed as previously described [15]. In brief, after cleaning, shaving and sterile preparation of the surgical field, a low midline laparotomy was performed, and a 2 × 5 cm^2^ full wall segment of the bladder was excised. The detrusor muscle and the mucosal layer were carefully separated mechanically with scissors and 2 × 3 cm^2^ from each of the resected layers were used for the tubular PLATE graft as described in the following section. For implantation, four horizontal incisions were made through the skin and subcutaneous fat, approximately 2 cm apart and laterally to the lower three mamillary papillae on the left side. Thereafter, the tubular constructs were sutured with two resorbable sutures (Vicryl™ 4 − 0, Ethicon, Johnson & Johnson, New Brunswick, US) on each end to the fascia of the shivering muscle (*panniculus carnosus)*. As the thickness of subcutaneous fat above the shivering muscle was macroscopically increasing in the cranial direction, the tubular constructs with and without muscle micrografts were implanted in an alternating fashion between subjects to mitigate any potential varying impacts on graft take and vascularization. The subcutaneous fat and skin was closed above the implanted tubes with interrupted non-resorbable sutures for later identification (Prolene™ 3 − 0, Ethicon, Johnson & Johnson, New Brunswick, US), and after discontinuation of anesthesia, the animals were housed, treated, and observed according to high standards for good animal practice and as previously described [15].

### Tubular graft construction

A detailed description of the PLATE graft methodology and construction can be found in a previous publication [20]. In brief, bladder mucosa and detrusor muscle were mechanically separated by sharp dissection and separately minced into approximately 1 mm^2^ micrografts and placed on each side of a 2 × 6 cm^2^ mesh (Vicryl™, cat#VM1208, Ethicon, Johnson & Johnson, New Brunswick, US) at a 1:6 expansion ratio (i.e., 1 cm^2^ of each excised tissue type was expanded onto a 6 cm^2^ mesh). In half of the constructs (urothelial group), the same amount of bladder mucosal micrografts were placed on one side, with no muscular micrografts being placed on the other side (collagen only), hereby serving as the control group. The expected degradation time of the Vicryl mesh in porcine tissue was 2–3 months [21]. After placement of the micrografts onto the mesh, the construct was embedded in liquid rat-tail collagen type 1 (2.06 mg/ml protein in 0.6% acetic acid, First Link Ltd, Wolverhampton, UK) in a steel mold and incubated for 5 min at 37 °C to solidify. Prior to embedding, the collagen had been diluted 4:1 with DMEM (Gibco, Thermo Fisher Scientific, Waltham, US) and pH-approximated to 7.4 with 1 M NaOH (Gibco, Thermo Fisher Scientific, Waltham, US). After solidification, the graft was then compressed with 250 g for 5 min to expel excess water. Finally, the PLATE graft was sutured around a 2 cm long sterile 14 Fr silicone catheter with interrupted sutures (PDS™ 4 − 0, Ethicon, Johnson & Johnson, New Brunswick, US) (Fig. 2A). Each tubular PLATE graft was 1 cm long (excess tube was used for anchoring to the subcutaneous fascia).

### Histology and immunoassay preparations

After termination of the animals, the tubular constructs were resected in toto with a native tissue margin of approximately 1 cm. After cutting one end of the resected tissue, the silicone catheters were manually removed with pincers and the specimens were fixed in 4% formalin for 24 h. Then, each construct was cut transversely in three to four equal sized pieces, before dehydration, paraffin embedding, and microtome sectioning into 5 μm sections, as per standard protocol. All tubular grafts from all twelve pigs were stained for routine histology with Hematoxylin & Eosin (H&E, CS70030-2 + CS70130-2, DAKO, Santa Clara, US), and a highly sensitive epithelium specific marker pancytokeratin CK-AE (Clone AE1/AE3, ID: GA053, DAKO, Agilent, Santa Clara, US). Additionally, selected representative grafts were stained for Desmin (Clone DE-R-11, Roche, Indianapolis, US) and uroplakin II (Clone BC21, ACI3051C, Biocare, Pacheco, US). All stained sections were scanned using a Visiopharm Oncotopix scanner (Hamamatsu, Shizuoka, JP) to perform digital quantification analyses of the sections.

### Measurement of histological graft thickness

To evaluate the graft thickness from the lumen to the surrounding subcutaneous adipose tissue, cross-sectional sections of pancytokeratin-stained specimens from all twelve pigs were analyzed using the NDP.view2© histology software (version 2.9.29 (RUO), Hamamatsu Phototonics K.K.). Using the *freehand region* function, the total areas of the grafts were morphologically identified and measured after conditional blinding of the assessor. In addition to the total cross-sectional area of the tubular grafts, the thickness from the luminal surface to the surrounding subcutaneous adipose tissue was also assessed. This was calculated as the mean thickness measured at 0°, 90°, 180°, and 270° relative to the graft center (supplementary Fig. 1).

### Immunofluorescence staining

Tissue sections from three of the twelve pigs were processed for immunofluorescence staining. First, the tissues were deparaffinized at 60 °C for 30 min before being placed in xylene followed by decreasing concentrations of ethanol. Heat-induced antigen retrieval was performed using a microwave with the tissue slides being placed in a 0.01 M citrate buffer (pH 6.0) at 95 °C for 15 min. The sections were then permeabilized using 0.5% triton 100X solution in phosphate-buffered saline (PBS) for 10 min. Subsequent serum blocking was performed by 10% normal donkey serum (17-000-121, Jackson ImmunoResearch Europe Ltd, Cambridge House, UK) and Bovine Serum Albumin (Product number A9418, Sigma Aldrich, Saint Louis, US) in PBS solution for 30 min. A mixture of the following primary antibodies was then applied at 4 °C overnight: rabbit anti-uroplakin II, 1:50 dilution (ab204756, Abcam, Cambridge Biomedical Campus, UK), goat anti-alpha-smooth muscle actin, 1:400 dilution, (NB300-978, Novus Biologicals, Centennial, US), and mouse anti-CD68, 1:50 dilution, (sc-20060, Santa Cruz Biotechnology Inc, Dallas, US). Similarly, goat anti-CD31 at 1:50 dilution (AF3628, R&D Systems, Inc, Abingdon, UK) was applied to individual slides from the same tissue specimens. The following day, the slides were incubated at room temperature for one hour with a mixture of the secondary antibodies: donkey anti-goat conjugated with Alexa Fluor 750 dye (Abcam, Cambridge Biomedical Campus, UK), donkey anti-mouse conjugated with Alexa Fluor 488 dye, (Thermo Fisher Scientific, Waltham, US), and donkey anti-rabbit conjugated with Alexa Fluor 647 (ab150075, Abcam, Cambridge Biomedical Campus, UK), all at 1:300 dilutions in PBS. For the slides stained with goat anti-CD31, only the donkey anti-goat secondary antibody was added. The tissues were counterstained using DAPI 1 mg/ml (Chemometec A/S, San Diego, US). A quenching kit (Vector TrueVIEW™ Autofluorescence Quenching Kit, VECTSP-8400-15, Vector Laboratories, Burlingame, US) was applied for 3 min to minimize mesh and collagen autofluorescence, according to manufacturer’s instructions. Finally, the slides were mounted using an aqueous mounting medium (Vectashield vibrance M, VECH-1700, Newark, US). Negative controls of the co-transplanted tissue sections were stained with secondary antibodies individually and can be seen in supplementary Fig. 2. The slides were scanned using the Zeiss Axioscan 7 slide scanner microscope with a x20 objective (Carl Zeiss, Oberkochen, DE).

### TUNEL assay

To evaluate apoptotic cells in the tissue, an in-situ cell death detection kit was used according to the manufacturer’s recommendations (cat#11684795910, Roche Life Sciences, Penzberg, DE). The kit fluorescently labels DNA strand breaks by an enzymatic reaction. Briefly, 5 μm sections of specimens from three pigs were deparaffinized as previously described. The sections were permeabilized using proteinase K for 30 min before being incubated with the TUNEL reaction mixture for two hours at 37 °C. The slides were subsequently counterstained using DAPI and mounted with a coverslip using a fluorescent mounting medium, and finally scanned as described in the previous section.

### Histological quantification

Prior to histological quantification and analysis of the slides, all files were blinded to the assessor by a non-involved colleague (EP in acknowledgement). A region of interest was manually annotated for analysis on each specimen using the QuPath software (version 0.5.0) [22]. The annotated region was selected as a 1 mm brim extending from the lumen of the constructs, before manually deleting areas where the constructs were visibly damaged during handling of the specimens, where the thickness of the grafts were visually thinner, or areas where the secondary antibodies appeared to not have been washed out properly (obvious staining artefacts). The number of immunofluorescence or TUNEL positive cells per mm^2^, respectively, were counted using a custom script for the StarDist plugin [23]. For the quantification of vessels in the grafts, vascular structures were manually counted using the QuPath software (version 0.5.0) after conditional blinding of the assessor (AGC).

### Statistical analysis

Results were stated as means with either standard deviations (SD) or 95% confidence intervals. Two-tailed independent t-tests were performed with *p* < 0.05 considered statistically significant. Statistical calculations were computed using Rstudio software (version 2023.12.0.36, PBC, Boston, MA, USA). Graphs were made using Plotly Chart Studio [24].

## Results

### Morphological assessment

All tubular grafts were successfully assembled and implanted subcutaneously as a single-staged procedure in all twelve minipigs as presented in Fig. 2A-C. All tubular grafts were removed after the planned 6-week study period. Of the 48 grafts in total, 7 grafts could not be processed for histological assessment, and were therefore excluded from further analysis. The non-included grafts were all inadvertently mechanically damaged due to misjudgment in placement. None of them showed any macroscopic signs of infection or increase in inflammation. This left a total of 19/24 and 22/24 grafts in the urothelial and co-transplanted group, respectively. No visible signs of infection or severe graft-host reactions were observed in any specimens at the time of graft removal and sectioning. In H&E staining, remnants of the mesh were still visible close to the center of the graft walls (Fig. 2D-E). The implanted micrografts could no longer be distinguished from the surrounding tissue. When measuring the total cross-sectional areas of the regenerated tubular graft walls, from the lumen to the adjacent subcutaneous tissue, there were no significant differences between the two groups (mean area of 17.3 ± 5.6 (SD) mm^2^ in the urothelial group vs. 14.7 ± 4.5 (SD) mm^2^ in the co-transplanted group, *p* = 0.13). Similarly, when assessing graft thickness from the luminal surface to the adjacent subcutaneous tissue based on measurements taken at 0°, 90°, 180°, and 270° relative to the tubular graft center, no significant difference was observed between the two groups (mean thickness 1.03 ± 0.25 (SD) mm in the urothelial group vs. 0.97 ± 0.22 (SD) mm in the co-transplanted group vs. *p* = 0.44).

### Immunohistochemical stains

To evaluate the presence of a luminal epithelium in the grafts, 2–4 technical replicates of each graft were stained for pancytokeratin (Fig. 2F). In these, 14/19 (74%) of the urothelial group, and 14/22 (64%) of the co-transplanted group had luminal pancytokeratin present in at least one of the technical replicates. There was no significant difference in the number of pancytokeratin positive grafts between the two groups (*p* = 0.524). To further assess the presence of cells of urothelial origin in the grafts, the grafts were stained for uroplakin II. As seen in Fig. 2G, uroplakin II was present luminally, but also scattered diffusely in more peripheral areas of the tissue (in contrast to native porcine bladder tissue, supplementary Fig. 3).

### Fluorescence microscopy

To further quantify and evaluate the distribution of α-SMA (marker of smooth muscle cells), uroplakin II (urothelial cells), CD31^+^ cells (blood vessels), and CD68^+^ cells (marker for macrophages), the tissues were analyzed with immunofluorescence. For evaluation of apoptotic cells, an enzymatic TUNEL assay was performed. α-SMA positive cells were observed abundantly throughout the graft wall in both experimental groups. CD68 and TUNEL-positive cells were primarily observed in the proximity of the mesh fibers as well as close to the graft lumen, where the cells were in direct contact with the silicone catheter. In accordance with the uroplakin II (Biocare, Pacheco, US) found in routine staining, as seen in Fig. 2G, the immunofluorescent uroplakin II (Abcam, Cambridge, UK) staining cells were diffusely dispersed throughout the graft wall, rather than being confined to the luminal, mucosal cells.

When evaluating the number of CD31 positive vessels per mm², no significant difference was observed between the two groups (19.2 ± 3.0 (SD) vessels per mm^2^ in the urothelial group vs. 18.5 ± 7.0 (SD) vessels per mm^2^ in the co-transplanted group, *p* = 0.83) (Fig. 3A). For comparison, the native bladder sections appeared more vascularized with a mean of 53.1 ± 33.5 (SD) vessels per mm^2^. No significant difference of uroplakin II positive cells was observed between the two groups (842 ± 682 (SD) cells per mm^2^ in the urothelial group vs. 1036 ± 959 (SD) cells per mm^2^ in the co-transplanted group, *p* = 0.70) (Fig. 3B). There was no significant difference observed between the two groups when comparing α-SMA positive cells (3305 ± 1542 (SD) cells per mm^2^ in the urothelial group vs. 3495 ± 2160 (SD) cells per mm^2^ in the co-transplanted group, *p* = 0.87) (Fig. 3C). Of the total number of positive α-SMA positive cells across both groups, a total of 15.1% of the α-SMA positive cells were also positive for uroplakin II. This is demonstrated in Fig. 4, where α-SMA & uroplakin II were often co-localized in the same cells. Stains for desmin, a protein found in smooth muscle cells but not in myofibroblasts [25], were performed on selected representative slides to further investigate the nature of the α-SMA positive cells. In contrast to α-SMA, desmin was only very scarcely seen in the tubular graft wall in both groups. Nonetheless, well-defined cell-bundles with high desmin concentrations were seen in the co-transplanted group located on the external side of the mesh, corresponding to the original location of the muscular micrografts (Fig. 5). These characteristic cell-bundles were not seen in the urothelial group.

When comparing the level of apoptotic cells by a TUNEL assay, no significant difference was observed (1086 ± 370 (SD) cells per mm^2^ in the urothelial group vs. 977 ± 670 (SD) cells per mm^2^ in the co-transplanted group, *p* = 0.78, Fig. 6A-B). Lastly, no significant difference in the number of CD68 positive cells was observed between the two groups (194 ± 136 (SD) cells per mm^2^ in the urothelial group vs. 179 ± 80 (SD) cells per mm^2^ in the co-transplanted group, *p* = 0.81, Fig. 6C). As expected, the number of CD68 positive cells and the number of apoptotic cells detected by the TUNEL assay was lower in the native bladder sections, with means (SD) of 27 (± 36) cells per mm^2^ and 7 (± 8) cells per mm^2^, respectively.

Negative controls of the secondary antibodies used to detect CD68, uroplakin-II and α-SMA positive cells revealed a faint signal in proximity of the mesh fibers in all three negative controls, interpreted as autofluorescence of the mesh (supplementary Fig. 2).

## Discussion

In this study, we evaluated the effects of co-transplanting bladder muscular micrografts with urothelial micrografts in a tubular collagen-based PLATE graft compared to transplanting urothelial micrografts only. By transplanting tubular grafts with and without muscular micrografts to the same pig, the pigs served as their own controls, allowing us to directly compare the effects of the added muscular micrografts. In a previous study, evaluating co-transplantation in a different setting, muscular micrografts were found to inhibit the expansion of epithelial micrografts when co-transplanted in a mixed fashion [12]. In the present study, where we embedded the muscular micrografts in a separate compartment in the PLATE graft, we found that co-transplanting muscular micrografts had no negative effects on the expression of cytokeratin, uroplakin II, CD68, and α-SMA-positive cells, as well as on the degree of vascularization or the level of apoptotic activity. These findings indicated that adding the muscular grafts to a separate compartment could be superior to co-transplantation in a mixed fashion in the same layer. These new findings could be important to optimize the PLATE methodology as it could be useful when attempting to reconstruct organs with complex anatomical compositions such as the bladder wall, where the biomechanical properties are crucial for organ function. In addition, we found well-defined desmin-rich cell-bundles in the co-transplanted group only, but no significant difference in the presence of α-SMA positive cells between the two groups. Further studies, including biomechanical tests, will be needed to determine whether adding muscular micrografts had any beneficial effect on the biomechanical and functional properties of the graft.

While the aim of the present study was to examine the effects of co-transplanting muscular micrografts with urothelial micrografts in a single-stage in vivo procedure, the effects of co-transplanting smooth muscle cells with urothelial cells in grafts *after* ex vivo cell expansion have been evaluated by others. Feng et al. used porcine acellular corpus spongiosum matrices as a biomaterial and, in two steps, seeded it with lingual keratinocytes either with or without adding a smooth muscle cell layer [26]. After 14 days of in vitro incubation, the scaffolds were used to create a neourethra in a rabbit model. The findings indicated that after the 6-month study period, the scaffolds containing both cell types were superior to scaffolds containing keratinocytes only, in respect to prevention of stricture formation and thickness of the epithelium. Different seeding techniques for in vitro co-culturing of urothelium and smooth muscle cells have also been evaluated. By culturing cells on small intestinal submucosa (SIS), Zhang et al. demonstrated that a layered approach, where the cells were seeded in two stages, or a sandwich approach, where the cells were seeded on either side of the SIS, were superior to seeding the cells directly as a mixed co-culture [27]. The results from these and other [28] in vitro studies support our findings, that interactions between smooth muscle cells and epithelial cells may occur when seeded imprudently onto a scaffold.

In the present study, a high number of α-SMA positive cells were observed in both groups, regardless of whether the initial graft composition included smooth muscle micrografts or not. Given their morphology, their widespread distribution extending both luminally and deeper into the tissue, and their presence in our control group with urothelial micrografts alone, these likely represent cells migrating either from the native surrounding tissue or from micrografts. The urothelial micrografts were composed not only of urothelium but also cells originating from the submucosa, as the urothelium was mechanically separated from the bladder biopsy, allowing a heterogenous submucosal cell composition to be transplanted within the graft. The observed cell population may therefore represent myofibroblasts, another contractile α-SMA positive cell. In areas where α-SMA was widely distributed, desmin, a protein present in smooth muscle cells but absent in myofibroblasts [25], was nearly undetectable, providing additional indications that the α-SMA-positive cells were myofibroblasts. Instead, small desmin positive bundles of cells were identified in some of the co-transplanted graft group, but never in the urothelial group (Fig. 5). As these groups of cells appear to be located on the external side of the mesh, similarly to where the detrusor muscle micrografts were placed at the initial tubular graft construction, these cells likely stemmed from the initial smooth muscle micrografts. Altogether, our findings suggested that smooth muscle micrografts could be successfully transplanted in the PLATE graft, and could survive as free grafts in the conditions of the current experiment, such as in the subcutaneous tissue. However, the results also indicated that the current experimental conditions with urothelial micrografts may preferentially promote the proliferation and expansion of myofibroblasts over smooth muscle cells. Myofibroblasts have previously been shown to play a role in wound healing by mediating wound contraction through contractile cytoplasmic elements [29]. Myofibroblasts are also highly active in secreting ECM proteins including collagen I, III, IV and the glycoprotein fibronectin [30]. Additionally, they contribute to the inflammatory response by secretion of cytokines and chemokines, including IL-6, IL-8, and matrix metalloproteinases [31, 32]. In combination, the activation of myofibroblasts is thought to play a key role in fibrosis formation [33]. Indeed, fibrosis formation and graft shrinkage at follow-up have been reported as complications in previous clinical trials using tissue engineered grafts to reconstruct urethral [34] and bladder wall defects [7]. In the present study, the degree of stenosis formation could not be evaluated due to the presence of the silicone catheter. Efforts to alter the function and prolonged activation of myofibroblasts in tissue engineered grafts has been a specific area of research.

The presence of myofibroblasts in our current experiment could origin from various sources. These include transformation of dormant or migrated fibroblasts to myofibroblasts through paracrine effects of inflammatory cells or alternatively from epithelial to mesenchymal transformation (EMT) of the micrografted urothelial cells [35]. TGF-β has been shown to be a central mediator of fibroblast to myofibroblast conversion, by activating downstream signaling pathways including the Smad pathway and the *wnt*/β-catenin pathway [30, 36]. As these pathways represent possible targets, Zhang et al. successfully inhibited tissue fibrosis in a tissue engineered urethroplasty by delivering a *wnt* pathway inhibitor in an electrospun collagen/polymer scaffold [37]. Similarly, Weisheng et al. demonstrated that infusing an acellular collagen scaffold with a modified VEGF protein to create a neo-urethra in a beagle model promoted tissue regeneration in the scaffold [38]. If increased tissue fibrosis, in combination with excessive myofibroblast activation, is seen in future PLATE graft experiments, we hypothesize that we could deliver bioactive molecules involved in the Smad- or *Wnt*/β-catenin pathways to prevent fibrosis in future long-term in vivo experiments.

In the current study, a large number of cells stained for both uroplakin II and α-SMA as seen in Fig. 4, where some cells appear to have the two proteins co-localized in the cytoplasm. This could be caused by unspecific immunofluorescence staining of the primary antibodies. However, a similar distribution of uroplakin II staining cells was found when using a primary antibody from another provider (*Abcam*,* Cambridge*,* UK* versus *Biocare*,* Pacheco*,* US*) and the non-specific staining was never found in sections from the native bladder. Furthermore, the stains appeared to be highly specific (supplementary Fig. 3). We therefore speculate whether the co-localization of the two proteins could be due to EMT, and that the cells were in a transitional state between the two phenotypes of urothelial cells and myofibroblasts.

Interestingly, in an experiment by Xia et al. [39], liver fibrosis was induced in mice by bile duct ligation. After 12 weeks, cells in the biliary epithelium co-expressed both α-SMA and cytokeratin-19, a biliary epithelium marker, while also actively producing collagen I, indicating that epithelial to myofibroblast transition play a role in liver fibrosis. EMT of tubular epithelial cells in the kidney has also been shown to be a key contributor in renal fibrosis [40]. Previously our group have examined differentially expressed (DE) microRNAs during bladder wound healing in rats compared to non- wounded rat bladders. Two of the 13 DE microRNAs that were identified in the study belong to the miR-200 family and have been shown to play a role in the regulation of EMT processes, indicating that EMT also occurs to some extent during normal bladder wound healing [41]. In the present study, EMT could be the source of the abundant myofibroblasts seen in the grafts and might also explain the large number of α-SMA positive cells in the grafts without detrusor muscle micrografts. As one of the hallmarks of EMT is the acquisition of migratory capacities of the differentiated epithelial cells [42], this could potentially explain the diffusely scattered uroplakin II positive cells detected away from the lumen in the immunohistochemical and immunofluorescent stains. However, as a recent Nature consensus statement regarding future research of EMT states [43], conclusions about EMT should not be based on a few molecular markers only, and as such our explanations remain speculative and a topic for future research.

There are a number of limitations to our study. First, the grafts were implanted in the subcutaneous adipose tissue rather than the native environment of the donor micrografts. As uroplakin II is present in the urothelial plaque, uroplakin II is mainly expressed by the terminally differentiated superficial umbrella cells [44, 45]. Since the luminal side of the PLATE graft was in direct contact with the silicone catheter, the superficial cells likely lacked the stimulus needed to drive uroplakin II expression. This may explain why luminal epithelization was not achieved in all grafts. This may also explain the profuse presence of myofibroblasts, that has not been observed in previous PLATE graft experiments, when PLATE grafts have been placed in their native environment, e.g. as a urinary conduit [15, 16]. Additionally, the elasticity of the silicone catheter, that kept the graft lumen patent, most likely exerted a perpendicular pressure on the luminal side of the graft, further creating an unfavorable environment for the urothelial cells. Nevertheless, for future clinical applications, where the PLATE graft may be applied, catheters should not be necessary. Instead, when used in tubular grafts, bio-degradable stents can keep the lumen from collapsing until the micrografts have expanded sufficiently [15, 16].

Another limitation of the present study was the observed autofluorescence from the mesh fibers. We attempted to mitigate this by using an autofluorescence quenching kit. However, autofluorescence was still observed in negative controls, leading to an inflated number of positive cells counted. We evaluate that this only had a minimal impact on the results, as the autofluorescence was likely present in the same degree in both the urothelial and co-transplanted group and was only observed in small amount in the negative controls. As a supplementary analysis, we attempted to adjust for this by subtracting the observed number of cells in the negative controls from the observed number of cells in the two experimental groups. After this adjustment, the differences between the two groups remained statistically insignificant.

## Conclusions

The results of our study suggest that in a perioperatively created collagen-based graft, muscular micrografts could be added to the graft without inhibiting the growth and expansion of co-transplanted urothelial micrografts. Our findings highlight the importance of embedding micrografts with different cell types into separate compartments of the autologous tubular tissue grafts. In future applications, where a muscular layer of the tissue engineered graft is desirable, this methodology could be a reasonable approach. Further experimental studies characterizing the effects of additional muscular micrografts are ongoing.

## Supplementary Information

Below is the link to the electronic supplementary material.

Supplementary Material 1

## Data Availability

Study data not presented in the publication can be made available upon request directly to the corresponding author.
