# Supplementary material for: Single-staged in vivo co-transplantation of autologous muscular and urothelial micrografts as a composite tissue tube for urogenital reconstruction
Source: Pediatr Surg Int. 2026 Jan 9;42(1):64. doi: 10.1007/s00383-025-06259-5 (PMC12789135; doi:10.1007/s00383-025-06259-5)
Supplement: Supplementary file 1 — Supplementary Material 1 [file 383_2025_6259_MOESM1_ESM.docx]

**Supplementary figure 1**

**Supplementary Figure 1:** **Measurement of graft cross-sectional area.** Scale bar indicates 1 mm. Using the NDP.view2 software, the area of the lumen as well as the total area of the graft + lumen was measured to determine the cross-sectional area of the graft. Graft tissue was morphologically distinguished from surrounding subcutaneous tissue by cell density and the hematoxylin staining intensity of the graft collagen fibers. Arrows indicate directions relative to the graft center, from which the thickness of the graft wall was measured.

**
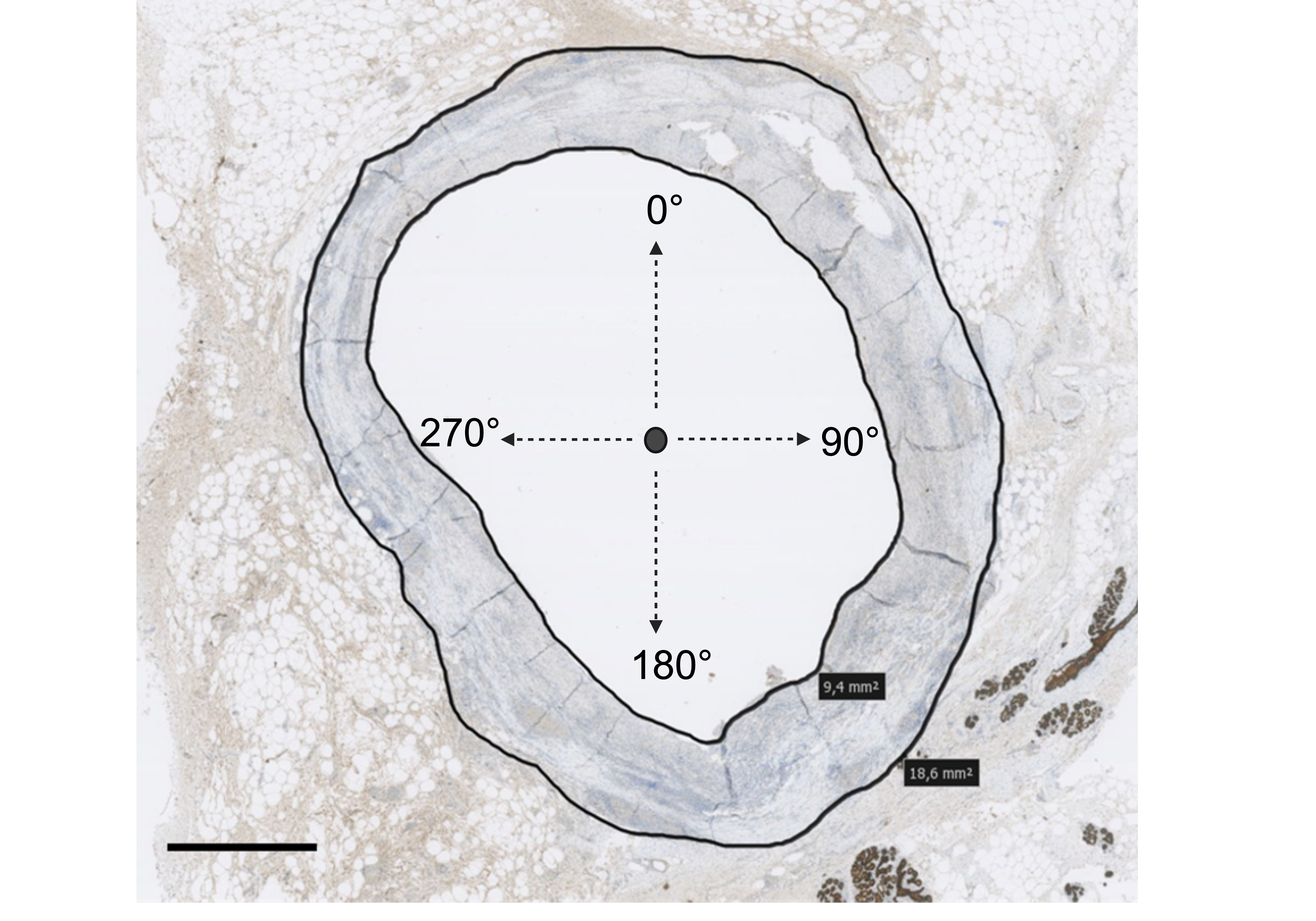
**


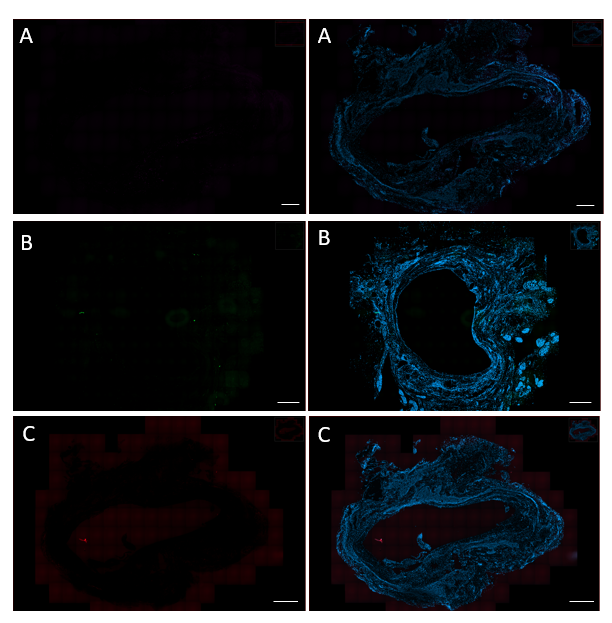
**Supplementary figure 2:**

***Supplementary figure 2: Immunofluorescence microscopy images of negative controls of secondary antibodies.*** *All stains are shown with and without DAPI nuclear staining and all stains were performed on co-transplanted grafts. A) Negative control of donkey anti-goat conjugated with Alexa Fluor 750 dye (used to stain anti-α-SMA & anti-CD31). Quantification of α-SMA-positive cells (performed on n=4 technical replicates), revealed a mean of 94 cells per mm^2^. Scale bars are 500 µm. B) Negative control of anti-mouse conjugated with Alexa Fluor 488 dye (used to stain anti-CD68). Quantification of CD68-positive cells (n=3) revealed 94 cells per mm^2^. Scale bars are 800 µm. C) Negative control of donkey anti-rabbit conjugated with Alexa Fluor 647 (used to stain anti-Uroplakin II). Quantifications of uroplakin II-positive cells (n=3) revealed 14 cells per mm^2^. Scale bars are 800 µm.*

**Supplementary figure 3:**
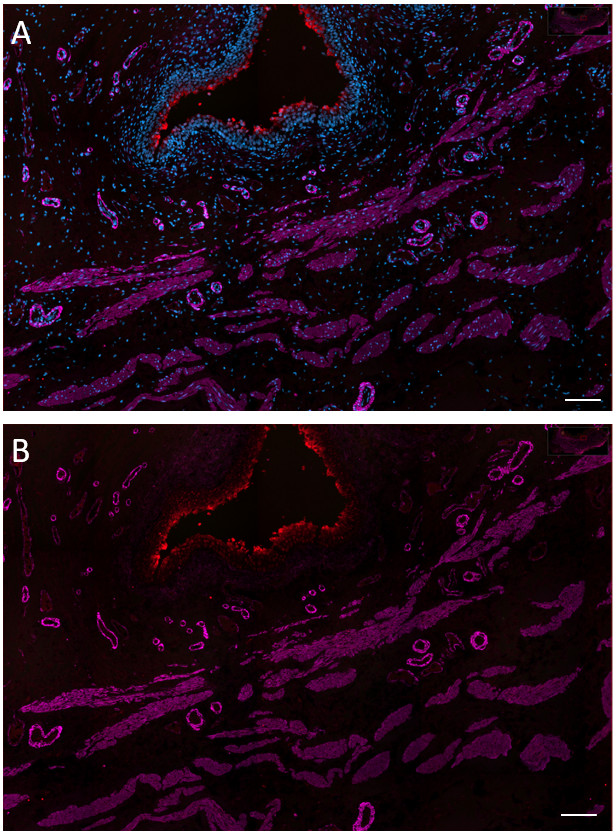


**Supplementary Figure 3: Immunofluorescence microscopy images of native bladder**. Scale bars indicate 100 µm. A) Immunofluorescence staining of α-SMA (pink) and Uroplakin II (red). Nuclear DAPI staining (blue) is also shown. The bladder lumen is visible towards the top of the image. B) The same image without the nuclear DAPI staining.
